# Editorial: Dietary supplements for preserving thyroid health: the scientific evidence-based view

**DOI:** 10.3389/fendo.2023.1213082

**Published:** 2023-06-13

**Authors:** Vittorio Unfer, Christian A. Koch, Salvatore Benvenga, Angela M. Leung

**Affiliations:** ^1^ Experts Group on Inositol in Basic and Clinical Research (EGOI), Rome, Italy; ^2^ UniCamillus-Saint Camillus International University of Health Sciences, Rome, Italy; ^3^ Department of Medicine, Fox Chase Cancer Center, Philadelphia, PA, United States; ^4^ Department of Medicine, University of Florida, Jacksonville, FL, United States; ^5^ University of Messina, Messina, Italy; ^6^ Division of Endocrinology, Diabetes, and Metabolism, Department of Medicine, University of California David Geffen School of Medicine, Los Angeles, CA, United States; ^7^ Division of Endocrinology, Diabetes, and Metabolism, Department of Medicine, VA Greater Los Angeles Healthcare System, Los Angeles, CA, United States

**Keywords:** thyroid disorders, dietary supplements, nutritional habits, thyroid health, euthyroidism, iodine, selenium

Thyroid disorders (TD) encompass a diverse array of diseases affecting thyroid anatomy or function, representing a global health problem. Among TD, hypothyroidism affects approximately 5% of the global population, and the percentage is increasing in recent years as the global population ages (1). Significant success was observed in the management of TD with the worldwide iodization of salt in the late 20th century, opening the door for a positive evaluation of dietary supplements to preserve thyroid health. Recognizing a need, the Research Topic ‘Dietary supplements for preserving thyroid health: the scientific evidence-based view’ invited contributors, totaling eight articles, highlighting recent developments on the role that dietary supplements, or nutraceuticals, play in TD.

To begin, a comprehensive review of the effects of trace elements in the thyroid is provided by Zhou et al.. In total, the authors cover 13 trace elements, compiling evidence on how thyroid volume, function, autoimmune thyroiditis, and thyroid carcinoma may be affected by high or low intake of those elements. The role of iodine and iron in modulating thyroid metabolism is discussed at length with iron deficiency causing a reduction of T3 and T4 levels, a reduction in peripheral T4-T3 conversion, and an overall increase in TSH levels. In addition, the authors speculate that prevention of trace element deficiency might not only have a positive effect on TD but also on the uptake of other trace elements.

Iodine levels in pregnant women are of particular interest as pregnancy is associated with an increase in TD (2). The major source of iodine worldwide is iodized salt (IS), therefore carefully balancing salt and iodine levels is of vital importance to preserve physiology in pregnancy. Sun et al. discuss the relationship between iodine sources and nutrition of pregnant women and adults, across four Chinese provinces. The authors observe that while iodine consumption in all four provinces was largely adequate, iodine consumption in the pregnant cohort was less than the recommended iodine intake (RNI) of 230 µg/day.

Continuing the theme of iodine supplementation, Gfeller et al. investigated the iodine contents in prenatal and adult multivitamins most commonly available in Switzerland. The authors report that, despite the importance of iodine supplementation during pregnancy and breastfeeding, only 14/23 (60.9%) of products indicated for women who were planning pregnancy, pregnant, or breastfeeding, contained iodine levels of ≥150 µg, with general use multivitamins performing worse. Taken together this suggests a need for greater levels of iodine in dietary supplements. Leung et al. (3) found that many supplements are mislabeled, not containing the iodine content that is stated on the label.

The next two articles focus on the use of myo-Inositol (myo-Ins) in TD, which gained much attention in recent years due to its endocrinological function in modulating insulin, FSH, and importantly TSH activity (4, 5). Paparo et al. discuss the use of myo-Ins in autoimmune thyroiditis reporting that myo-Ins administration, combined with selenium induces immuno-modulatory effects by reducing levels of thyroid antibodies, pro-inflammatory chemokines, and oxidative stress. Furthermore, a deficiency in myo-Ins was associated with increased cancer risk, while myo-Ins and selenium supplementation reduced thyroid nodule size, number, and elasticity (6).

The Research Topic then shifts the scope to focus on myo-Ins in combination with selenium in women with subclinical hypothyroidism. Payer et al. conducted a multicenter study in Slovakian women of reproductive age, where treatment of myo-Ins (600 mg) with selenium (63 mg) over 6 months resulted in a significant reduction of TSH levels, cholesterol, and the index of autoimmunity in a subset of the trial. Further significant improvements were seen in the regulation of the menstrual cycle in addition to the patient’s perception of symptoms. Interestingly, significant improvements in the parameters evaluated in the trial were observed already after 3 months of treatment.

Iodine uptake is thought to be affected by other nutrients, such as various vitamins, thus establishing an association between these and TD. Therefore, Capriello et al. present a minireview discussing the link between TD and vitamin A deficiency, reporting that vitamin A deficiency correlates with a reduction of iodine uptake and impaired iodothyronine coupling in the thyroid. In addition, several peripheral effects were observed, such as a strong reduction of hepatic T4 to T3 conversion. The research paper concludes by highlighting the potential use of vitamin A derivatives in thyroid cancer, as success has been reported in human thyroid cancer cell lines, with treatment increasing iodine uptake and sodium-iodine symporter activity (7, 8).

Continuing the theme of vitamin deficiency, Benites-Zapata et al. conducted a meta-analysis that included 64 different studies to investigate vitamin B12 levels in relation to TD. The authors describe an overall reduction of B12 levels in patients with hypothyroidism, with 25% of patients exhibiting B12 deficiency, while no significant level of B12 deficiency was observed between healthy subjects or those with hyperthyroidism. Those findings confirm the data from prior reviews, which stated 10-45% of patients with hypothyroidism exhibited a B12 deficiency, with the meta-analysis offering a much larger sample size with less variance and a higher degree of reliability.

In addition to dietary factors, other lifestyle choices such as exercise or smoking status are known to influence TD, therefore this Research Topic concludes with a retrospective cross-sectional study of thyroid nodules and their causes in Chinese adult women. Dong et al. report that risk factors across the sampled population include advanced age (50+) and manual labor, while protective factors include dietary diversity and normal triglyceride levels, suggesting a need for greater variation in the diet for the most at-risk groups.

The findings discussed in this Research Topic demonstrate the great potential of dietary supplements for TD. While iodine and selenium consumption remain central to treating and preventing TD, it is clear that other nutraceuticals such as myo-Ins, vitamin A, and vitamin B12, in addition to other trace elements have a role to play. It is also evident from the research that as an understanding of new supplement opportunities increases so must our knowledge of the target populations, to properly identify the at-risk groups for TD (9, 10). We, therefore, believe that these studies offer important contributions paving the way for further studies to counteract the challenging pathologies of the thyroid gland.

## Author contributions

VU-original draft preparation, review, and editing. All authors have read and agreed to the published version of the manuscript.
